# Brain Injury and Neurodegeneration: Molecular, Functional, and Translational Approach

**DOI:** 10.3390/biomedicines11071947

**Published:** 2023-07-10

**Authors:** Meenakshi Ahluwalia, Pankaj Gaur, Kumar Vaibhav

**Affiliations:** 1 Department of Pathology, Medical College of Georgia, Augusta University, Augusta, GA 30912, USA; mahluwalia@augusta.edu; 2 Georgia Cancer Center, Medical College of Georgia, Augusta University, Augusta, GA 30912, USA; 3 Department of Neurosurgery, Medical College of Georgia, Augusta University, Augusta, GA 30912, USA; 4 Lombardi Comprehensive Cancer Center, Georgetown University Medical Center, Washington, DC 20057, USA; pg684@georgetown.edu; 5 Department of Oral Biology and Diagnostic Sciences, Center for Excellence in Research, Scholarship and Innovation, Dental College of Georgia, Augusta University, Augusta, GA 30912, USA; 6 Transdisciplinary Research Initiative in Inflammaging and Brain Aging (TRIBA), Augusta University, Augusta, GA 30912, USA

Recently, we have achieved substantial progress in our understanding of brain injury and neurodegeneration. We have enhanced our knowledge about different brain pathologies or insults (accidental or non-accidental), such as hemorrhages, traumatic brain injury (TBI), ischemia, hypoxic/hypobaric insults, and neurological disorders such as Parkinson’s disease and Alzheimer’s disease (PD and AD). Brain pathology is multifactorial, encompassing a cascade of inflammation, necrotic, and apoptotic pathways. It is well known that brain insults or injuries to the brain may lead to neurological disorders over time, and genetic or environmental factors play essential roles in the progression of a brain disease. The absence of a specific cure to limit injury progression after an insult has persuaded the scientific community to study the mechanisms behind brain injury and degenerative cascade and to explore different therapeutic strategies.

This Special Issue, entitled “Brain Injury and Neurodegeneration: Molecular, Functional, and Translational Approach”, has addressed various important aspects of brain injury and neurodegeneration, such as TBI, cerebral hypoxia, epilepsy, AD, and SARS-CoV-2-mediated brain damage. This Special Issue has received an enthusiastic response globally, resulting in the publication of 18 peer-reviewed articles, including 10 research articles and 8 reviews.

The recent SARS-CoV-2 pandemic caused more than 3 million deaths globally [1]. Similarly to past coronavirus outbreaks, including SARS and MERS, SARS-CoV-2 infections were associated with fever, dry cough, pneumonia, fatigue, and acute respiratory distress syndrome (ARDS) [2]. However, each host–pathogen interaction leaves a footprint on the health of different organs [3]. Clinical studies on COVID-19 patients have revealed atypical symptoms and neurological signs, including headaches, anosmia, nausea, dysgeusia, damage to respiratory centers, and cerebral infarction [2,4]. Extreme cytokines release (cytokine storm) occurs due to aberrant immune pathways, and microglial activation propagates CNS damage in COVID-19 patients [1,2,3,4]. Moreover, elderly with neurological problems such as PD and AD showed a higher incidence of COVID-19-associated complications [4]. In this Special Issue, an Editor’s Choice article by Donegani et al. [5] provided proof of CNS damage via an [18F]-FDG PET scan. Twenty-two SARS-CoV-2 patients underwent whole-body [18F]-FDG PET examination, including a dedicated brain acquisition between May and December 2020 after their recovery from SARS-CoV-2 infection, and fourteen patients were found to have persistent hyposmia in bilateral fusiform gyri and parahippocampal, and in left insula, as compared to the controls [5].

Our Special Issue has a large number of TBI-related articles, which includes five research articles and one review. TBI is a global health concern as it results in substantial death and disability [6,7,8,9,10,11]. In light of this, our group [12] has provided a comprehensive review on TBI. In this Editor’s Choice review, we have provided a broad understanding of TBI pathology, mechanisms, inflammation, and immune interactions. Understanding these mechanisms and exploring potential targets for neuroprotective treatments are crucial for advancing new therapies. This review delves into the molecular events that occur following TBI, encompassing inflammation and programmed cell death, and offers an overview of the current literature and therapeutic approaches, contributing to a deeper understanding of secondary injuries caused by TBI. Cheng et al. [13] have shown that stretch injury (an in vitro model of TBI) in SH-SY5Y neuroblastoma cells altered the mitochondrial membrane potential and triggered oxidative DNA damage at 24 h. Stretch injury increased neuronal stress via reducing brain-derived neurotrophic factors (BDNFs) and increasing amyloid-β. Mechanistically, neuronal injury was exaggerated through the loss of the insulin pathway and via increased glycogen synthase kinase 3β (GSK-3β)S9/p-Tau protein levels [13]. In addition, Puhakka et al. [14] reported that small non-coding RNAs (sncRNAs) play a crucial role in modulating post-TBI neuroinflammation. Further, increased expression of the miR-146a profile and of 3′tRF-IleAAT and 3′tRF-LysTTT was found to be associated with behavioral deficits in animals with chronic TBI-induced neuroinflammation [14]. Another study by Vorn et al. [15] profiled the plasma exosomal microRNAs from young adults with mild TBI and from healthy individuals, and identified 25 dysregulated exosomal miRNAs in the chronic mTBI group 4.48 mean years after injury. These miRNAs are associated with pathways of neurological disease, organismal injury and abnormalities, and psychological disease, and can be useful to be diagnostic markers for chronic mTBI [15].

A higher percentage of patients with TBI die from secondary pathological processes despite the application of preventative measures and the provision of medical supervision [8,9,10,11]. Post-traumatic epilepsy (PTE) is one of the most common debilitating implications of TBI [16]. Post-traumatic seizures (PTS) are experienced in increasing TBI individuals who also display resistance to traditional anti-seizure medications (ASMs). Hentig et al. [17] identified an upregulated sonic hedgehog (Shh) signaling pathway in zebrafish after CNS injury that helps in regeneration. Shh signaling increases excitatory amino acid transporters (Eaat)2a to inhibit TBI-induced glutamate excitotoxicity and subsequent seizure sequelae [17]. Further, Ghosh et al. [18] provided an account for epilepsy, the roles played by various neurotransmitters and their corresponding receptors in the pathophysiology of epilepsy. One of the forefront areas of epilepsy research is drug-resistant epilepsy (DRE), which is the focus of this review. The authors mentioned that drug-resistant epilepsy (DRE) remains a prominent focus of research due to its link to psychosocial complications and premature mortality, and to a high prevalence among epileptic patients [18]. The review examines various hypotheses relating to DRE and explores unconventional therapeutic strategies and combination therapy. Additionally, recent studies supporting modern treatment approaches for epilepsy are discussed, with specific emphasis on the mTOR pathway, blood–brain barrier breakdown, and inflammatory pathways.

There are other factors, CNS insults and diseases, such as hypoxia, alcohol, viral infections, ischemia, and neurodegeneration, which contribute towards acute and chronic deficits in CNS function. Our collection also highlights the interesting findings in these particular areas. Both chronic alcoholism and human herpesvirus-6 (HHV-6) infection may cause movement-related disorders and promote neuroinflammation. Jain et al. [19] observed decreased perivascular CD68^+^/Iba1^+^ microglia in the postmortem brain from alcoholic individuals as compared to the dominant CD68^+^/Iba1^−^ microglial subpopulation in the control brains. All the control brains were HHV-6 negative. Further, HHV-6 infection in alcoholics elevated microglial dystrophic changes with higher Iba1^+^ cells and compounded the microglia-mediated neuroinflammation. Another research article by Baltanas et al. [20] explored the rare, biallelic variants of the AGTPBP1 gene that caused its loss of function, and led to childhood-onset neurodegeneration with cerebellar atrophy (CONDCA). Mutations in AGTPBP1 led to the substantial loss of cerebellar Purkinje cells in the mouse model of cerebellar ataxia, and might be used for CONDCA modeling in mice. Asik et al. [21] provided a review of Alzheimer’s disease in relation to Amyloid-β (Aβ). They have provided a comprehensive view of Amyloid-β (Aβ)-related pathology and mechanisms and of the current clinical status of anti-amyloid therapy. They emphasized that the relationship between dysfunctional mitochondrial and the progression of AD required further research. Yoshida et al. [22] reported that higher oxidative stress in hippocampal mitochondria leads to cognitive impairment in a 5xFAD mice model of Alzheimer’s disease. They further mentioned that age can be a vital factor in elevated oxidative stress in AD pathology, and preventing mitochondrial oxidative damage may be important to protect cognitive function.

Hypoxia as a result of the deprivation of oxygen, temporary or chronic, can either be adaptive or pathological. A systematic review by Stoica et al. [23] utilized the “Preferred Reporting Items for Systematic Reviews and Meta-Analyses (PRISMA)” filtering method, and probed five internationally renowned medical databases. Using this method, they identified 45 eligible papers and provided information on pathophysiology, mechanisms, and consequent clinical conditions following hypoxic episodes. Ischemia occurs as a result of transient or permanent interruption blood supply in a given region, which leads to poor oxygenation, inflammation, and oxidative stress. Fatty acid-binding proteins (FABPs) mediate lipid metabolism and regulate the dynamics of fatty acids. Following transient MCAO, the levels of FABP3, FABP5, and FABP7 were found to be upregulated in the brain. Guo et al. [24] reported that the FABP inhibitor, i.e., FABP ligand 6 [4-(2-(5-(2-chlorophenyl)-1-(4-isopropylphenyl)-1H-pyrazol-3-yl)-4-fluorophenoxy)butanoic acid] (referred to here as MF6), minimized the prostaglandin E2 (PGE2)-mediated inflammation in ischemic brain. Flavonoids, icariin (ICA), and icaritin (ICT) derived from *Herba epimedii* have been identified as neuroprotective phytochemicals. Wu et al. [25] reported that both ICA and ICT treatment improved neuronal cell apoptosis, minimized oxidative stress, and countered extracellular matrix (ECM) accumulation in mice brains post-acute cerebral ischemia. Demyanenko et al. [26] reviewed the role of histone deacetylases and their inhibitors in ischemic stroke.. The authors showed that ischemic stroke generally reduces gene expression via suppression of the acetylation of histones H3 and H4. Inhibitors to histone deacetylases promoted functional recovery post-cerebral ischemia by inducing neurogenesis and angiogenesis in the injured areas of brain. This review aimed to explore neuroprotective activities of histone deacetylase inhibitors in ischemic stroke. In line with neuroprotection, Haider et al. [27] investigated the role of mitoquinone in chronic neuroprotection post-TBI, and found that mitoquinone reduced gliosis, decreased oxidative stress, limited neuroinflamamtion, and improved axonal integrity and neuronal survival in an open-head CCI mouse model of moderate TBI.

With the increase in the expectancy of the life span of humans, incidences of accidental injury and neurodegenerative diseases (NDs) have risen and have imposed a considerable burden on the family, society, and the nation. The review by Khan et al. [28] explores the mechanisms of action of the phytochemicals and nutraceuticals available to date for various NDs. The group has reviewed clinical and pre-clinical studies involving phytochemicals in neurodegeneration. Despite phytochemicals showing a robust effect in animal studies, mixed results were observed in several clinical trials, and therefore, the authors stressed a need to reassess their efficacies in more robust clinical studies [28]. While traditional medicines and chemical inhibitors are being actively studied in pre-clinical and clinical settings, non-invasive methods such as exercise [29,30], whole-body vibration [31], or ischemic conditioning [32,33,34] can enhance endogenous protection against many diseases. Nhu et al. [35] reviewed treadmill exercise (TE) on neural mitochondria in PD. Parkinson’s disease is the second most common neurodegenerative disorder [36,37], and TE has been widely applied in its rehabilitation [30,38]. For this systematic review [35], the CAMARADES checklist was used to assess the methodological quality of the studies. The review findings supported the hypothesis that treadmill exercise could attenuate neuronal mitochondrial dysregulation and respiratory deficiency in PD and could slow down the progression of PD.

In conclusion, this Special Issue represents a novel and exciting perspective on brain injury, hypoxia, ischemia, and neurodegeneration, and it represents a comprehensive research resource for readers on disease-related pathology, mechanisms, and translational approach. However, we acknowledge that this field of neuroscience is under active research, and several new findings are being made daily. Therefore, this Special Issue of articles, along with new discoveries, will be an interesting and substantial read for scholars in this field.

## References

[1] Ahluwalia P., Vaibhav K., Ahluwalia M., Mondal A.K., Sahajpal N., Rojiani A.M., Kolhe R. (2021). Infection and Immune Memory: Variables in Robust Protection by Vaccines Against SARS-CoV-2. Front. Immunol..

[2] Jarrahi A., Ahluwalia M., Khodadadi H., da Silva Lopes Salles E., Kolhe R., Hess D.C., Vale F., Kumar M., Baban B., Vaibhav K. (2020). Neurological consequences of COVID-19: What have we learned and where do we go from here?. J. Neuroinflamm..

[3] Ahluwalia P., Ahluwalia M., Vaibhav K., Mondal A., Sahajpal N., Islam S., Fulzele S., Kota V., Dhandapani K., Baban B. (2020). Infections of the lung: A predictive, preventive and personalized perspective through the lens of evolution, the emergence of SARS-CoV-2 and its pathogenesis. EPMA J..

[4] Hsu P.C., Shahed-Al-Mahmud M. (2022). SARS-CoV-2 mediated neurological disorders in COVID-19: Measuring the pathophysiology and immune response. Life Sci..

[5] Donegani M.I., Miceli A., Pardini M., Bauckneht M., Chiola S., Pennone M., Marini C., Massa F., Raffa S., Ferrarazzo G. (2021). Brain Metabolic Correlates of Persistent Olfactory Dysfunction after SARS-Cov2 Infection. Biomedicines.

[6] Coronado V.G., Xu L., Basavaraju S.V., McGuire L.C., Wald M.M., Faul M.D., Guzman B.R., Hemphill J.D. (2011). Surveillance for traumatic brain injury-related deaths—United States, 1997–2007. Morb. Mortal. Wkly. Rep. Surveill. Summ..

[7] Stevens J.A., Corso P.S., Finkelstein E.A., Miller T.R. (2006). The costs of fatal and non-fatal falls among older adults. Inj. Prev. J. Int. Soc. Child Adolesc. Inj. Prev..

[8] Bramlett H.M., Dietrich W.D. (2002). Quantitative structural changes in white and gray matter 1 year following traumatic brain injury in rats. Acta Neuropathol..

[9] Bramlett H.M., Dietrich W.D. (2007). Progressive damage after brain and spinal cord injury: Pathomechanisms and treatment strategies. Prog. Brain Res..

[10] Taylor C.A., Bell J.M., Breiding M.J., Xu L. (2017). Traumatic Brain Injury-Related Emergency Department Visits, Hospitalizations, and Deaths—United States, 2007 and 2013. Morb. Mortal. Wkly. Rep. Surveill. Summ..

[11] Destounis A., Tountas C., Theodosis-Georgilas A., Zahos P., Kasinos N., Palios J., Beldekos D. (2019). An unusual case of double-chambered left ventricle: A case of double-chambered left ventricle communicated with right ventricle through a ventricular septal defect presented during only in diastole and a concomitant mitral valve prolapse. J. Echocardiogr..

[12] Jarrahi A., Braun M., Ahluwalia M., Gupta R.V., Wilson M., Munie S., Ahluwalia P., Vender J.R., Vale F.L., Dhandapani K.M. (2020). Revisiting Traumatic Brain Injury: From Molecular Mechanisms to Therapeutic Interventions. Biomedicines.

[13] Cheng P.W., Wu Y.C., Wong T.Y., Sun G.C., Tseng C.J. (2021). Mechanical Stretching-Induced Traumatic Brain Injury Is Mediated by the Formation of GSK-3β-Tau Complex to Impair Insulin Signaling Transduction. Biomedicines.

[14] Puhakka N., Das Gupta S., Vuokila N., Pitkänen A. (2022). Transfer RNA-Derived Fragments and isomiRs Are Novel Components of Chronic TBI-Induced Neuropathology. Biomedicines.

[15] Vorn R., Suarez M., White J.C., Martin C.A., Kim H.S., Lai C., Yun S.J., Gill J.M., Lee H. (2021). Exosomal microRNA Differential Expression in Plasma of Young Adults with Chronic Mild Traumatic Brain Injury and Healthy Control. Biomedicines.

[16] Yu T., Liu X., Sun L., Wu J., Wang Q. (2021). Clinical characteristics of post-traumatic epilepsy and the factors affecting the latency of PTE. BMC Neurol..

[17] Hentig J., Campbell L.J., Cloghessy K., Lee M., Boggess W., Hyde D.R. (2021). Prophylactic Activation of Shh Signaling Attenuates TBI-Induced Seizures in Zebrafish by Modulating Glutamate Excitotoxicity through Eaat2a. Biomedicines.

[18] Ghosh S., Sinha J.K., Khan T., Devaraju K.S., Singh P., Vaibhav K., Gaur P. (2021). Pharmacological and Therapeutic Approaches in the Treatment of Epilepsy. Biomedicines.

[19] Jain N., Smirnovs M., Strojeva S., Murovska M., Skuja S. (2021). Chronic Alcoholism and HHV-6 Infection Synergistically Promote Neuroinflammatory Microglial Phenotypes in the Substantia Nigra of the Adult Human Brain. Biomedicines.

[20] Baltanás F.C., Berciano M.T., Santos E., Lafarga M. (2021). The Childhood-Onset Neurodegeneration with Cerebellar Atrophy (CONDCA) Disease Caused by AGTPBP1 Gene Mutations: The Purkinje Cell Degeneration Mouse as an Animal Model for the Study of this Human Disease. Biomedicines.

[21] Mohamed Asik R., Suganthy N., Aarifa M.A., Kumar A., Szigeti K., Mathe D., Gulyás B., Archunan G., Padmanabhan P. (2021). Alzheimer’s Disease: A Molecular View of β-Amyloid Induced Morbific Events. Biomedicines.

[22] Yoshida N., Kato Y., Takatsu H., Fukui K. (2022). Relationship between Cognitive Dysfunction and Age-Related Variability in Oxidative Markers in Isolated Mitochondria of Alzheimer’s Disease Transgenic Mouse Brains. Biomedicines.

[23] Stoica S.I., Bleotu C., Ciobanu V., Ionescu A.M., Albadi I., Onose G., Munteanu C. (2022). Considerations about Hypoxic Changes in Neuraxis Tissue Injuries and Recovery. Biomedicines.

[24] Guo Q., Kawahata I., Degawa T., Ikeda-Matsuo Y., Sun M., Han F., Fukunaga K. (2021). Fatty Acid-Binding Proteins Aggravate Cerebral Ischemia-Reperfusion Injury in Mice. Biomedicines.

[25] Wu C.T., Chen M.C., Liu S.H., Yang T.H., Long L.H., Guan S.S., Chen C.M. (2021). Bioactive Flavonoids Icaritin and Icariin Protect against Cerebral Ischemia-Reperfusion-Associated Apoptosis and Extracellular Matrix Accumulation in an Ischemic Stroke Mouse Model. Biomedicines.

[26] Demyanenko S., Dzreyan V., Sharifulina S. (2021). Histone Deacetylases and Their Isoform-Specific Inhibitors in Ischemic Stroke. Biomedicines.

[27] Haidar M.A., Shakkour Z., Barsa C., Tabet M., Mekhjian S., Darwish H., Goli M., Shear D., Pandya J.D., Mechref Y. (2022). Mitoquinone Helps Combat the Neurological, Cognitive, and Molecular Consequences of Open Head Traumatic Brain Injury at Chronic Time Point. Biomedicines.

[28] Khan A., Jahan S., Imtiyaz Z., Alshahrani S., Antar Makeen H., Mohammed Alshehri B., Kumar A., Arafah A., Rehman M.U. (2020). Neuroprotection: Targeting Multiple Pathways by Naturally Occurring Phytochemicals. Biomedicines.

[29] Khan M.B., Alam H., Siddiqui S., Shaikh M.F., Sharma A., Rehman A., Baban B., Arbab A.S., Hess D.C. (2023). Exercise Improves Cerebral Blood Flow and Functional Outcomes in an Experimental Mouse Model of Vascular Cognitive Impairment and Dementia (VCID). Transl. Stroke Res..

[30] Wang R., Tian H., Guo D., Tian Q., Yao T., Kong X. (2020). Impacts of exercise intervention on various diseases in rats. J. Sport Health Sci..

[31] Yin H., Berdel H.O., Moore D., Davis F., Liu J., Mozaffari M., Yu J.C., Baban B. (2015). Whole body vibration therapy: A novel potential treatment for type 2 diabetes mellitus. Springerplus.

[32] Khan M.B., Hoda M.N., Vaibhav K., Giri S., Wang P., Waller J.L., Ergul A., Dhandapani K.M., Fagan S.C., Hess D.C. (2015). Remote ischemic postconditioning: Harnessing endogenous protection in a murine model of vascular cognitive impairment. Transl. Stroke Res..

[33] Vaibhav K., Braun M., Khan M.B., Fatima S., Saad N., Shankar A., Khan Z.T., Harris R.B.S., Yang Q., Huo Y. (2018). Remote ischemic post-conditioning promotes hematoma resolution via AMPK-dependent immune regulation. J. Exp. Med..

[34] Hess D.C., Khan M.B., Kamat P., Vaibhav K., Dhandapani K.M., Baban B., Waller J.L., Hoda M.N., Blauenfeldt R.A., Andersen G. (2021). Conditioning medicine for ischemic and hemorrhagic stroke. Cond. Med..

[35] Nhu N.T., Cheng Y.J., Lee S.D. (2021). Effects of Treadmill Exercise on Neural Mitochondrial Functions in Parkinson’s Disease: A Systematic Review of Animal Studies. Biomedicines.

[36] Kalia L.V., Lang A.E. (2015). Parkinson’s disease. Lancet.

[37] Sveinbjornsdottir S. (2016). The clinical symptoms of Parkinson’s disease. J. Neurochem..

[38] Mehrholz J., Kugler J., Storch A., Pohl M., Elsner B., Hirsch K. (2015). Treadmill training for patients with Parkinson’s disease. Cochrane Database Syst. Rev..

